# Alginate Hydrogel-Embedded Capillary Sensor for Quantitative Immunoassay with Naked Eye

**DOI:** 10.3390/s20174831

**Published:** 2020-08-27

**Authors:** Wenshu Zheng, Cen Gao, Liheng Shen, Chang Qu, Xuan Zhang, Lu Yang, Qiang Feng, Rongbing Tang

**Affiliations:** 1 School of Stomatology, Lanzhou University, Lanzhou 730000, China; gaoc15@lzu.edu.cn (C.G.); shenlh14@lzu.edu.cn (L.S.); quchang3313@mails.jlu.edu.cn (C.Q.); xuanzhang18@lzu.edu.cn (X.Z.); yanglu19@lzu.edu.cn (L.Y.); 2 National Center for Nanoscience and Nanotechnology, University of Chinese Academy of Sciences, Beijing 100190, China; wzheng5@tulane.edu (W.Z.); qiang.feng@utsouthwestern.edu (Q.F.); 3 School of Medicine, Tulane University, New Orleans, LA 70112, USA; 4 Department of Pharmacology Harold C. Simmons Comprehensive Cancer Center, University of Texas Southwestern Medical Center, TX 75390, USA

**Keywords:** immunoassay, POCT, alginate hydrogel, capillary microfluidics

## Abstract

We have developed an alginate hydrogel-embedded capillary sensor (AHCS) for naked eye-based quantification of immunoassay. Alkaline phosphatase (ALP) can modulate gel-sol transformation to increase the permeability of Cu^2+^-cross-linked alginate hydrogel film in the AHCS, followed by solution exchange into the capillary. Through measuring the length of the liquid phase of the microfluidics in the capillary at a given time, the concentration of the ALP could be quantified with the naked eye. Since ALP is widely applied as a signal reporter for immunoassays, the AHCS could easily accommodate conventional immune sensing platforms. We justify the practicality of AHCS with hepatitis B virus surface antigen (HBsAg) in serum samples and got comparable results with commercialized immunoassay. This AHCS is easy to make and use, effective in cost, and robust in quantification with the naked eye, showing great promise for next generation point-of-care testing.

## 1. Introduction

During the pandemic of infectious diseases, efficient tests can significantly slow down the spread of diseases and improve public health decision-making. Decentralizing the complex biomarker assay from laboratory sites to remote areas and temporary stations with sample-to-answer point-of-care-test (POCT) devices will greatly improve the testing efficiency [1,2,3,4]. Antibody-based immunoassays have achieved extraordinary success in the sensing of biomarkers during the past for more than half a century [5,6]. However, the gold standard method for immunoassay, enzyme-linked immunosorbent assay (ELISA), as a highly sensitive platform, relies on specialized spectroscopic instruments to record absorbance for quantification, which is impossible to use outside professional labs [7]. Although lateral flow assay (LTFA) is a very successful easy-to-use POCT detection platform, the poor sensitivity and non-quantifiable result limit its use to only a few special scenarios [8,9,10].

Tremendous efforts have been implemented for improving the performance of POCT platforms [11,12,13], among which the stimuli-responsive hydrogels achieved broad popularity [14]. Good biocompatibility and unique mechanical properties have enabled diverse application for hydrogels, including drug delivery, contaminant separation, and chemical and biological sensing [15,16,17,18,19,20]. In particular, hydrogel can be designed to swell/collapse in the presence of analytes by gel/sol phase transformation, which provides a visual signal that enables naked eye-based readout without expensive instruments. Based on this principle, hydrogel-based assays have been developed for the sensing of metal ions [21,22], glucose [23], metabolites [24], pH [25], and biomolecules [26]. However, to achieve sensitive response to targets, hydrogels are customized according to individual purposes with complicated chemical synthesis and modifications [27]. After those modifications, the hydrogel can only be respond to one pre-set target based on the target recognition components and is not available for the sensing of other analytes. Developing a global applicable hydrogel system remains challenging in the field; however, it may be an ideal platform for convenient POCT with the following criteria: (i) the sensitivity is compared to traditional ELISA, (ii) easy to prepare and cost-effective, (iii) achieve qualitative or even quantitative detection, and without any instrument, (iv) could be easily modified for sensing different types of biomarkers.

We have developed an alginate hydrogel-embedded capillary sensor (AHCS) for naked eye quantification of immunoassay. The Cu^2+^-cross-linked alginate hydrogel is reported to be PPi-responsive and has been incorporated with carbon nanodots to enable effective fluorescent quantification of alkaline phosphatase (ALP) under fluorescent spectroscopy [28]. In addition, in order to avoid the reliance on special instruments, several visual sensors have been recently developed where target-induced gel-sol transformation can modulate the permeability of the hydrogel, and further influence the capillary behavior to provide a distance-based signal in the capillary for visual readout [29,30]. Inspired by these findings, we designed a distance-based sensor for ALP and immunoassays with the naked eye. In our approach, the target is detected with alkaline phosphatase (ALP)-labeled antibody through the immune recognition process, during which the amount of target will eventually affect the amount of ALP. The ALP will further hydrolyze PPi and inhibit the phase transformation inside AHCS, where Cu^2+^-cross-linked alginate hydrogels are optimized for highly sensitive gel-sol transition in the presence of pyrophosphate ion (PPi). The dissolved gel will be exchanged by the outside water phase solution after the successful phase transformation. As a result, the amount of the target finally determines the length of liquid flow in AHCS at a given time due to the capillary action. We justify the practicability of AHCS by the detection HBsAg in human seru samples. Since the flow distance could be easily observed with the naked eye, our assay would enable the rapid, quantitative sensing of biomarkers without the reliance on any instruments, and is suitable as a routine POCT device for disease diagnosis, especially in remote areas and temporary testing stations.

## 2. Materials and Methods

### 2.1. Reagents

All chemicals used in this study were ordered from major suppliers (Sigma-Aldrich, St. Louis, MO, USA, ACS grade) unless otherwise noted, including CuCl_2_, KCl, NaCl, Sodium Alginate, Sodium pyrophosphate tetrabasic, human serum albumin (HSA), lysozyme, thrombin, and glucose oxidase (GOx). Human hepatitis B virus surface antigen ELISA kit (EY-00H428) is from Shanghai Yiyan Culture Communication Co., Ltd. (Shanghai, China) capillary tube (0.3 mm) is from Shanghai Great wall Company (Shanghai, China).

### 2.2. Preparation of Cu-Cross-Linked Hydrogel

To generate the Cu-cross-linked hydrogel for sensing the ALP and immunoassay, we mixed the solutions of alginate with different concentration of Cu^2+^. The mixtures were placed at an incubator shaker heated at 60 °C for 10 min, and then cooled down to room temperature to grantee the homogeneity of the hydrogel. The prepared hydrogel was immediately used for the fabrication of AHCS.

### 2.3. Fabrication of AHCS for Visual Immunoassay

We washed the capillary tubes with piranha solution, acetone, methanol, and deionized water under ultrasonic in a sequence, and then dried with nitrogen gas. To form the hydrogel film at the end of the capillary tube. We placed the capillary tube vertically on the surface of the heated hydrogel (60 °C) for 5 s. We chose the capillary tube with a hydrogel film inside the tube with the same thickness (0.3 mm) for further measurements.

### 2.4. Sensing of ALP with AHCS

We mixed a 20-μL mixture reaction buffer with PPi (2 mM) and a different concentration of ALP for 30 min incubation at room temperature, and then immersed one end of the AHCS in the mixture for 30 min. We recorded the length of liquid flow in the AHCS using smartphone ruler software or a physical ruler.

### 2.5. Selectivity of AHCS

We mixed a 20-μL mixture reaction buffer with PPi (2 mM) and different concentrations of interferences, including Na^+^, K^+^, human serum albumin (HSA), lysozyme, thrombin, and glucose oxidase (GOx) with a final concentration of 1 mM for 30 min incubation at room temperature, and then immersed the AHCS in the mixture for 30 min. The length of liquid flow in the AHCS was then recorded.

### 2.6. AHCS for Immune Sensing of Hepatitis B Virus Surface Antigen (HBsAg)

We added different samples to each well of the 96-well plate that was coated with capture antibody against HBsAg and blocked with Bovine Serum Albumin (BSA). After 1 h incubation at 37 °C, the plates were washed with an additional PBST (0.01 M PBS 0.5% Tween-20) for 3 times, and 100 μL solution of diluted (1 μg/mL) biotin-conjugated mouse anti-Human HBsAg antibody (100 μL) was added, and further incubated at 37 °C for 1 h. The plates were washed with PBST for an additional three times, and streptavidin-conjugated ALP (1 μg/mL) was added to the plates for 1 h at 37 °C. After another three rounds of washing, we added 2 mM PPi to the plate for 30 min incubation at room temperature and then used AHCS for the sensing.

### 2.7. Traditional ELISA for Immune Sensing of HBsAg

The procedure for detecting HBsAg by ELISA was basically the same as the procedure for detecting HBsAg by AHCS, except, after adding ALP-conjugate anti-HBsAg antibody, ALP substrate (para-Nitrophenylphosphate, 100 μL) instead of PPi was added to the mixture. A microplate reader was used to record the absorbance from the mixtures.

## 3. Results

### 3.1. Principle of ALP-Modulated Gel-Sol Transformation

**Figure 1:**
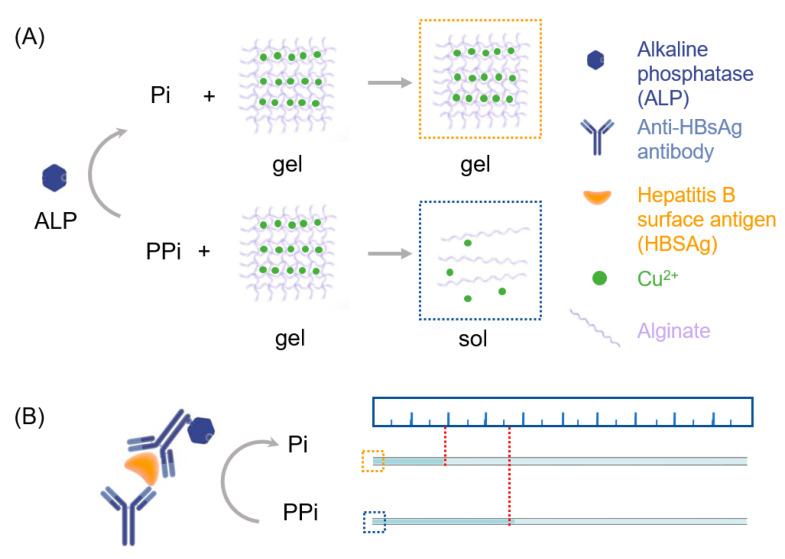
Scheme of AHCS as a quantitative immunoassay. (**A**) The principle of alkaline phosphatase (ALP)-triggered sol-gel transition of Cu^2+^ cross-linked-alginate hydrogel. (**B**) Principle of AHCS-based immunoassay using ALP-labeled antibody.

Alginate has been well established to form hydrogels after chelating with Cu^2+^. We prepared the Cu^2+^-cross-linked hydrogel to react with the mixture containing PPi and different concentrations of ALP. As Cu^2+^ can form stable complex due to the very high stability constant between Cu^2+^ and PPi [31], In the absence of ALP, more PPi would be competitively binding with Cu^2+^ to disrupt the Cu^2+^-cross-linked alginate hydrogel and induce a fast gel-sol transformation. By contrast, in the presence of ALP, the PPi would be hydrolyzed to phosphate ions (Pi), since Pi has a lower affinity with Cu^2+^, and the gel-sol transformation will be inhibited (Figure 1A). The degree of gel-sol transformation would influence the permeability of the hydrogel film, and further influence the speed of the reaction mixture to flow into the capillary tube. As a result, the amount of ALP will determine the distance of the flow, which can be easily observed with the naked eye and quantified with a ruler. Since ALP is widely applied as a signal reporter for immunoassays, the AHCS could easily accommodate conventional immune sensing platforms that employs ALP for signal generation (Figure 1B).

### 3.2. Optimization of Cu^2+^ in AHCS for ALP Detection

**Figure 2:**
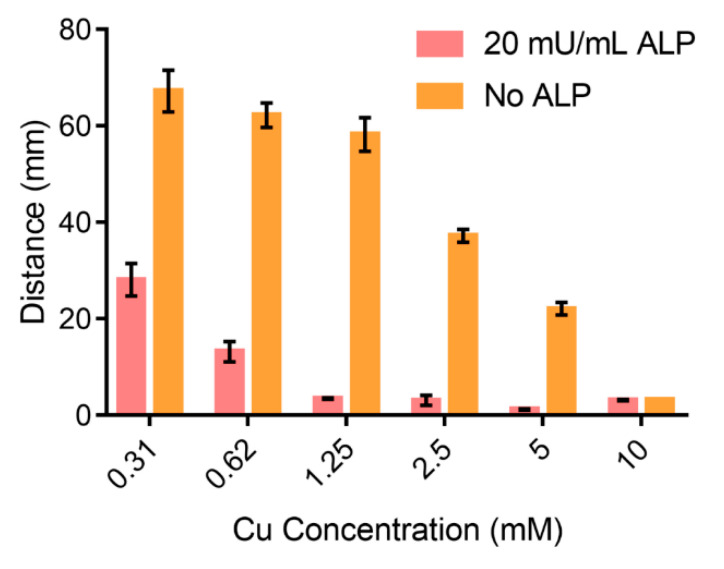
Distance of the flow in AHCS responding to different concentration of Cu.

We optimized the concentration of Cu^2+^ to enable the most sensitive response towards ALP. Even though the high concentration of Cu^2+^ could increase the crosslinking degree and the stability of the alginate hydrogel (Figure S1 in Supplementary Materials), these hydrogel may become less sensitive to the concentration of PPi. More PPi would be required to trigger the gel-sol transformation to influence the permeability of the hydrogel film. We mixed 50 mU/mL ALP and 2 mM PPi for 30 min at room temperature and then applied AHCS prepared with different concentration of Cu^2+^ for the sensing of ALP (Figure 2). AHCS prepared with a higher concentration (10 mM) of Cu^2+^ could not show gel-sol response to the PPi despite the presence of ALP. As a comparison, ALP could not totally suppress the gel-sol transformation for AHCS prepared with lower concentration (0.31 mM) of Cu^2+^. When the AHCS is prepared with 1.25 mM of Cu^2+^, the largest change in the flow distance (45.23 ± 2.53 mm) between the ALP + PPi group and the PPi group could be observed in 30 min. We, thus, chose AHCS prepared with 1.25 mM of Cu^2+^ for our further study.

### 3.3. Sensitivity of AHCS for ALP Detection

**Figure 3:**
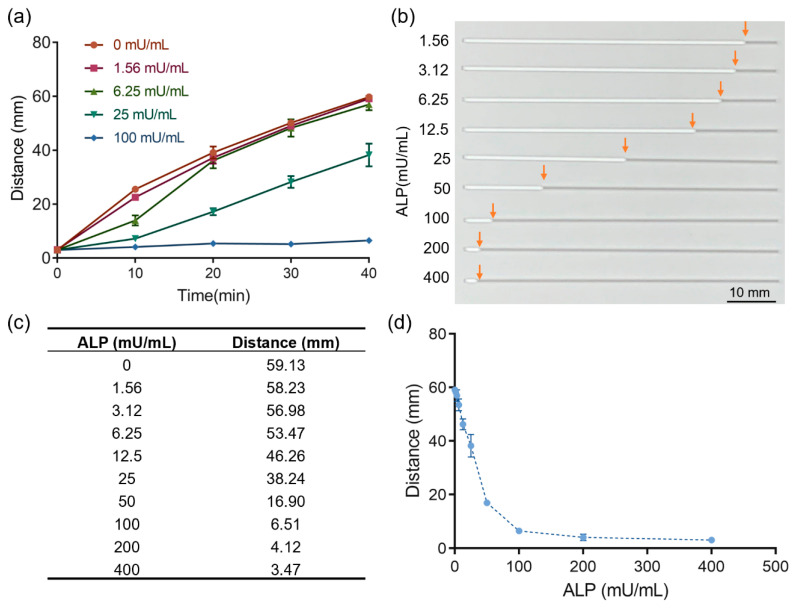
Alginate hydrogel-embedded capillary sensor (AHCS) for the detection of ALP. (**a**) Time-distance dependent to different concentration of ALP measured with AHCS. (**b**) Photos of AHCS responding to different concentration of ALP. The white color in the capillary is gel and gray color in the capillary is air. (**c**) Distance of the flow in AHCS responding to different concentration of ALP. (**d**) The relationship of distance to different concentrations of ALP. Mean ± SD, n = 3.

We tested the response of AHCS to different concentration of ALP. ALP could catalyze PPi to Pi and suppress the gel-sol transition, which further decreased the permeability of hydrogel film and liquid exchange into the capillary tube. As shown by the readout of AHCS, the flow rate of the sample in the capillary gradually increased when the concentration ALP concentration increased (Figure 3a). The distance of liquid phase flow at 30 min became shorter when the concentration of ALP increased from 0 mU/mL to 200 mU/mL (Figure 3b–d). The difference of distance can be observed when the concentration of ALP was above 3.12 mU/mL with the naked eye (Figure 3b,c). When the concentration of ALP was higher than 100 mU/mL, the PPi was completely hydrolyzed to suppress the gel-sol transformation, resulting in a very small distance of flow in the capillary tube (Figure 3d). We used a ruler to measure the flow distance induced by the samples that contained different concentrations of ALP after 30 min. We got a linear response following the equation: distance (mm) = −5.272 × ALP (mU/mL) + 58.85 (R^2^ = 0.995), when the ALP concentration was from 1.56 mU/mL to 50 mU/mL (Figure S2 in Supplementary Materials). The result shows the potential of AHCS for quantitative sensing of ALP.

### 3.4. Sensitivity of AHCS for Immunoassay

**Figure 4:**
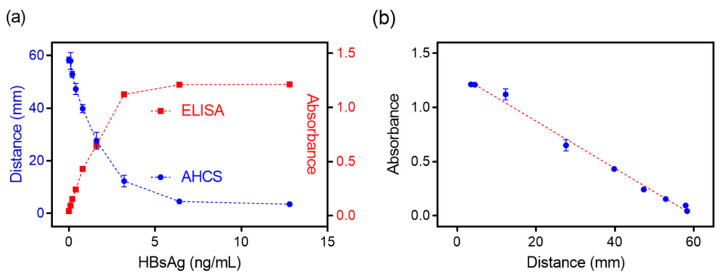
Comparable result was obtained with AHCS and ELISA for immunoassay. (**a**) The relationship of the distance measured with ELISA or distance measured with AHCS to different concentrations of HBsAg using AHCS with ALP-labeled goat-anti-human antibody as the detection Antibody. (**b**) The relationship of the absorbance measured with ELISA to distance measured with AHCS. Mean ± SD, n = 3.

We applied AHCS in sensing antigen using ALP-labeled antibody, which is a widely used method in the current ELISA platform. We used hepatitis B surface antigen (HBsAg) as a model target to verify the capability of AHCS for immunoassay. In the presence of the HBsAg, ALP-labeled antibody was conjugated on the plates via immune recognition to trigger the hydrolysis of PPi to Pi, which further inhibited the sol-gel transformation to influence the distance of flow. We observed clear differences in the distance of flow in the capillary when the concentration of HBsAg was as low as 0.3 ng/mL with the naked eye. By measuring the distance of flow induced by the samples that contained different concentration of HBsAg after 30 min, a linear response between the flow distance and the concentration of HBsAg is observed following the equation: distance (mm) = −9.494 × Con. (HBsAg (ng/mL)) + 57.29 (R^2^ = 0.9831), and the LOD is 0.24 ng/mL using AHCS (Figure 4a and Figure S3 in Supplementary Materials). Commercially available HBsAg ELISA with the readout from a plate reader shows a LOD (0.114 ng/mL) and liner response (OD value = 0.3238 × Con. (HBsAg (ng/mL)) + 0.1091, R^2^ = 0.9896) (Figure 4a and Figure S4 in Supplementary Materials). As a comparison, AHCS enables the quantitatively immunoassay without relying on plate reader (Figure 4b), which is suitable for developing point-of-care, and applied in remote areas and temporary stations.

### 3.5. The Selectivity of AHCS for Immunoassay

**Figure 5:**
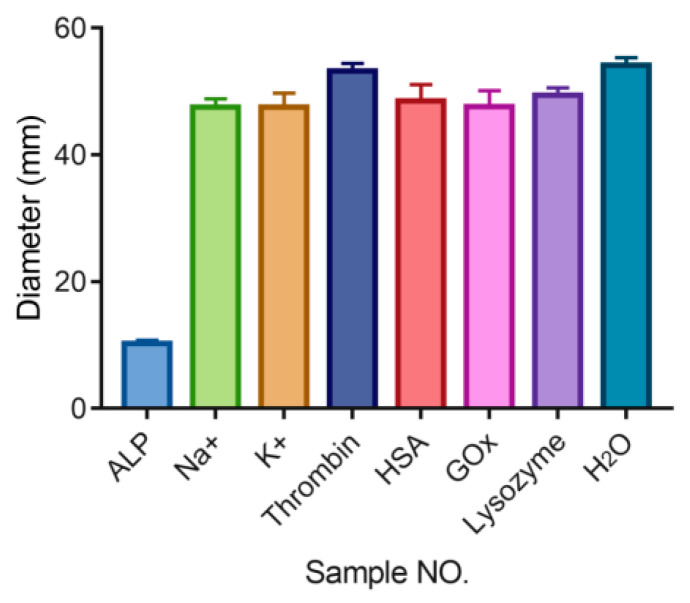
Flow distance measured with AHCS in the presence of different interference, The ALP concentration is 100 mU/mL. The concentrations of other interferences are 1 mM. Mean ± SD, n = 3.

We tested the possible interference in biological samples that may influence the immunoassay with AHCS (Figure 5). We used AHCS to sense several interferences under the same protocol of sensing ALP, including Na^+^, K^+^, human serum albumin (HSA), lysozyme, thrombin, and glucose oxidase (GOx). Even though the concentration of these interferences (1 mM) is much higher than the concentration of ALP (100 mU/mL). There are no significant differences in the flow distances in response to these interferences compared to that towards H_2_O, suggesting that these interferences would neither hydrolyze the PPi to prevent gel-sol transformation nor directly influence the structure of the Cu^2+^-crosslinked alginate hydrogel. The high selectivity of our assay enables AHCS to be further applied for immune sensing in clinical samples.

### 3.6. AHCS for Immunoassay in Human Serum Samples

**Figure 6:**
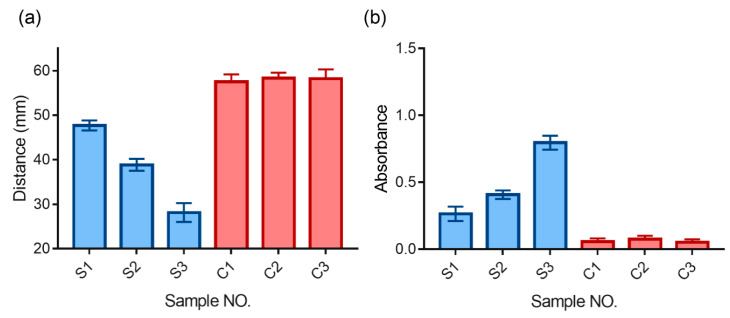
Quantitative detection of hepatitis B surface antigen (HBsAg) in serum samples. (**a**) AHCS and its comparison with traditional ELISA (**b**). Mean ± SD, n = 3.

Knowing the high specificity and sensitivity for AHCS, we test the real sample application of AHCS for immunoassay in HBsAg-spiked serum samples from healthy donor. Different concentrations of HBsAg were added to the serum from a healthy donor (S1, S2, S3) and quantified with either AHCS or tradition ELIAS. Neither AHCS nor traditional could detect the presence of HBsAg from un-spiked health donor (C1, C2, C3). Compared to control samples, both AHCS and ELISA show the strongest response to S3, and the weakest response to S1, suggesting the good consistency for the detection performance between AHCS (Figure 6a) and ELISA (Figure 6b). Table 1 shows the added concentration and the quantified results from AHCS or traditional ELISA; AHCS shows a recovery of 91.55~93.2% for samples S1, S2, and S3, which is also comparable to traditional ELISA (92.1~95.7%). AHCS is a robust sensing platform for immunoassay for naked eye based clinical use.

## 4. Discussion

We provide a portable sensor for immunoassays based on ALP-triggered gel-sol transformation of alginate-hydrogel. The transformation will change the permeability of the hydrogel film and modulate the liquid exchange, which enables distance-based quantitative sensing with the naked eye. Our method is compatible for the detection of HBsAg in human serum with high reproductivity, as a proof-of-concept example. Compared with traditional immunoassay, AHCS shows comparable sensitivity, linear response, and quantitative quality with naked eye readout. In addition, AHCS could easily accommodate current immunoassays by employing different ALP labeled antibodies. Further efforts will focus on the automated, multiple samples processing, and analyzing. We envision AHPS would become a robust analytic tool for point-of-care testing for biomarkers, especially in resource-limited areas and temporary stations.

## Figures and Tables

**Table 1:** Quantitative result of HBsAg in serum samples using AHCS or traditional ELISA.

Sample No.	Added HBsAg (ng/mL)	AHCS (ng/mL)	ELISA (ng/mL)
S1	0.5	0.4621	0.4785
S2	1	1.0945	0.9212
S3	2	1.864	2.123

## Supplementary Materials

The following are available online at https://www.mdpi.com/1424-8220/20/17/4831/s1, Figure S1. Photos of Cu^2+^-crosslinked alginate hydrogel. Figure S2. Linear response for ALP using AHCS. Figure S3. Linear response for HBsAg using AHCS. Figure S4. Linear response for HBsAg using commercialized ELISA.

## References

[1] Huang R., Zhang K., Zhu G., Sun Z., He S., Chen W. (2018). Blocking-Free ELISA using a gold nanoparticle layer coated commercial microwell plate. Sensors.

[2] Zheng W., Li H., Chen W., Ji J., Jiang X. (2016). Recyclable Colorimetric Detection of Trivalent Cations in Aqueous Media Using Zwitterionic Gold Nanoparticles. Anal. Chem..

[3] Cánovas R., Cuartero M., Crespo G.A. (2019). Modern creatinine (Bio) sensing: Challenges of point-of-care platforms. Biosens. Bioelectron..

[4] Broughton J.P., Deng X., Yu G., Fasching C.L., Servellita V., Singh J., Miao X., Streithorst J.A., Granados A., Sotomayor-Gonzalez A. (2020). CRISPR–Cas12-based detection of SARS-CoV-2. Nat. Biotechnol..

[5] Gan S.D., Patel K.R. (2013). Enzyme immunoassay and enzyme-linked immunosorbent assay. J. Investig. Dermatol..

[6] Zheng W., Zeng L., Chen Y. (2020). Bioorthogonal Reactions Amplify Magnetic Nanoparticles Binding and Assembly for Ultrasensitive Magnetic Resonance Sensing. Anal. Chem..

[7] De La Rica R., Stevens M.M. (2012). Plasmonic ELISA for the ultrasensitive detection of disease biomarkers with the naked eye. Nat. Nanotech..

[8] Sajid M., Kawde A.-N., Daud M. (2015). Designs, formats and applications of lateral flow assay: A literature review. J. Saudi Chem. Soc..

[9] Choi D.H., Lee S.K., Oh Y.K., Bae B.W., Lee S.D., Kim S., Shin Y.-B., Kim M.-G. (2010). A dual gold nanoparticle conjugate-based lateral flow assay (LFA) method for the analysis of troponin I. Biosens. Bioelectron..

[10] Taton K., Johnson D., Guire P., Lange E., Tondra M. (2009). Lateral flow immunoassay using magnetoresistive sensors. J. Magn. Magn. Mater..

[11] Dutta S. (2019). Point of care sensing and biosensing using ambient light sensor of smartphone: Critical review. TrAC Trends Anal. Chem..

[12] Chen W., Li Q., Zheng W., Hu F., Zhang G., Wang Z., Zhang D., Jiang X. (2014). Identification of bacteria in water by a fluorescent array. Angew. Chem. Int. Ed..

[13] Xu L., Lu Z., Cao L., Pang H., Zhang Q., Fu Y., Xiong Y., Li Y., Wang X., Wang J. (2017). In-field detection of multiple pathogenic bacteria in food products using a portable fluorescent biosensing system. Food Control..

[14] Deng Z., Guo Y., Zhao X., Ma P.X., Guo B. (2018). Multifunctional stimuli-responsive hydrogels with self-healing, high conductivity, and rapid recovery through host–guest interactions. Chem. Mater..

[15] Kahn J.S., Hu Y., Willner I. (2017). Stimuli-responsive DNA-based hydrogels: From basic principles to applications. Acc. Chem. Res..

[16] Lei Z., Wang Q., Sun S., Zhu W., Wu P. (2017). A bioinspired mineral hydrogel as a self-healable, mechanically adaptable ionic skin for highly sensitive pressure sensing. Adv. Mater..

[17] Tokarev I., Minko S. (2010). Stimuli-responsive porous hydrogels at interfaces for molecular filtration, separation, controlled release, and gating in capsules and membranes. Adv. Mater..

[18] Wang K., Burban J., Cussler E. (1993). Hydrogels as separation agents. Responsive Gels: Volume Transitions II.

[19] Lee A.L., Voo Z.X., Chin W., Ono R.J., Yang C., Gao S., Hedrick J.L., Yang Y.Y. (2018). Injectable coacervate hydrogel for delivery of anticancer drug-loaded nanoparticles in vivo. ACS Appl. Mater. Interfaces.

[20] Ozay O., Ekici S., Baran Y., Aktas N., Sahiner N. (2009). Removal of toxic metal ions with magnetic hydrogels. Water Res..

[21] Gogoi N., Barooah M., Majumdar G., Chowdhury D. (2015). Carbon dots rooted agarose hydrogel hybrid platform for optical detection and separation of heavy metal ions. ACS Appl. Mater. Interfaces.

[22] Ye B.-F., Zhao Y.-J., Cheng Y., Li T.-T., Xie Z.-Y., Zhao X.-W., Gu Z.-Z. (2012). Colorimetric photonic hydrogel aptasensor for the screening of heavy metal ions. Nanoscale.

[23] Ma Y., Mao Y., An Y., Tian T., Zhang H., Yan J., Zhu Z., Yang C.J. (2018). Target-responsive DNA hydrogel for non-enzymatic and visual detection of glucose. Analyst.

[24] Liu R., Huang Y., Ma Y., Jia S., Gao M., Li J., Zhang H., Xu D., Wu M., Chen Y. (2015). Design and synthesis of target-responsive aptamer-cross-linked hydrogel for visual quantitative detection of ochratoxin A. ACS Appl. Mater. Interfaces.

[25] Griffete N., Frederich H., Maître A., Ravaine S., Chehimi M.M., Mangeney C. (2012). Inverse opals of molecularly imprinted hydrogels for the detection of bisphenol A and pH sensing. Langmuir.

[26] Peppas N.A., Van Blarcom D.S. (2016). Hydrogel-based biosensors and sensing devices for drug delivery. J. Control. Release.

[27] Zhang J., Mou L., Jiang X. (2018). Hydrogels incorporating Au@ polydopamine nanoparticles: Robust performance for optical sensing. Anal. Chem..

[28] Li Y., Huang Z., Weng Y., Tan H. (2019). Pyrophosphate ion-responsive alginate hydrogel as an effective fluorescent sensing platform for alkaline phosphatase detection. Chem. Commun..

[29] Jiang C., Li Y., Wang H., Chen D., Wen Y. (2020). A portable visual capillary sensor based on functional DNA crosslinked hydrogel for point-of-care detection of lead ion. Sens. Actuators B Chem..

[30] Li Y., Ma Y., Jiao X., Li T., Lv Z., Yang C.J., Zhang X., Wen Y. (2019). Control of capillary behavior through target-responsive hydrogel permeability alteration for sensitive visual quantitative detection. Nat. Commun..

[31] Chen C., Zhao D., Sun J., Yang X. (2016). Colorimetric logic gate for pyrophosphate and pyrophosphatase via regulating the catalytic capability of horseradish peroxidase. ACS Appl. Mater. Interfaces.

