# Supplementary material for: Alginate Hydrogel-Embedded Capillary Sensor for Quantitative Immunoassay with Naked Eye
Source: Sensors (Basel). 2020 Aug 27;20(17):4831. doi: 10.3390/s20174831 (PMC7506732; doi:10.3390/s20174831)
Supplement: Supplementary file 1 [file sensors-20-04831-s001.pdf]

Supplementary Materials For:

Alginate Hydrogel-Embedded Capillary Sensor for Quantitative Immunoassay with Naked Eye

Wenshu Zheng [1,2,3,†], Cen Gao [1,†], Liheng Shen [1], Chang Qu [1], Xuan Zhang [1], Lu Yang [1], Qiang Feng [2,4] and Rongbing Tang [1,*]

[1] School of Stomatology, Lanzhou University, Lanzhou 730000, China; wzheng5@tulane.edu (W.Z.); gaoc15@lzu.edu.cn (C.G.); shenlh14@lzu.edu.cn (L.S.); quchang3313@mails.jlu.edu.cn (C.Q.); xuanzhang18@lzu.edu.cn (X.Z.); yanglu19@lzu.edu.cn (L.Y.)

[2] National Center for Nanoscience and Nanotechnology, University of Chinese Academy of Sciences, Beijing 100190, China; qiang.feng@utsouthwestern.edu

[3] School of Medicine, Tulane University, New Orleans, LA 70112, USA

[4] Department of Pharmacology Harold C. Simmons Comprehensive Cancer Center, University of Texas Southwestern Medical Center, TX 75390, USA

* Correspondence: tangrb@lzu.edu.cn

† These authors contributed equally to this work.

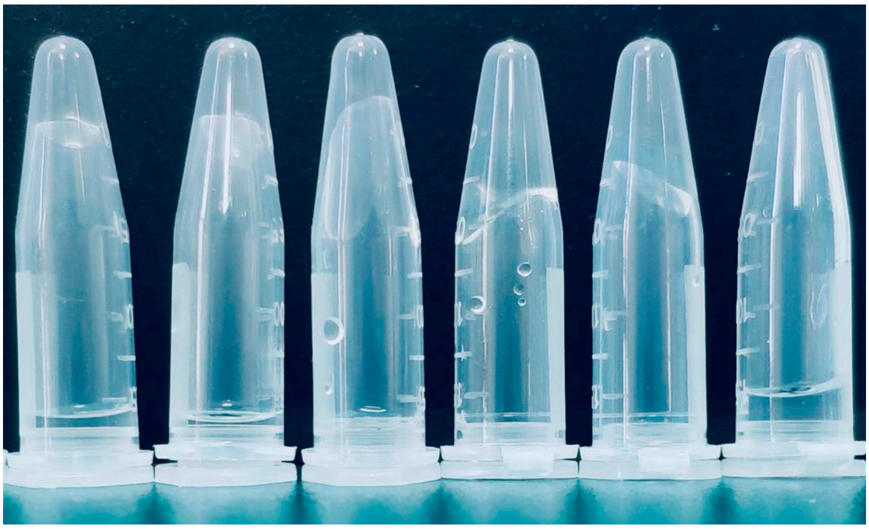

Figure S1. Photos of alginate hydrogel crosslinked with different concentration (from left to right 0.5 mM, 1 mM, 2 mM, 4 mM, 8 mM, 16 mM, 0) of $CuCl_2$

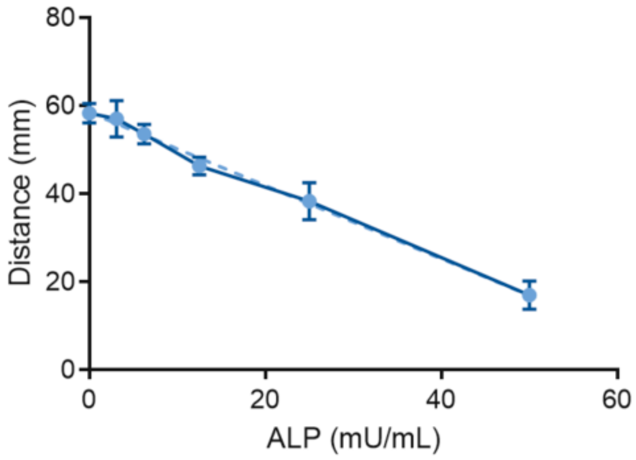

Figure S2. Linear response between the flow distance and the concentration of ALP using AHCS, mean+SD, n=3

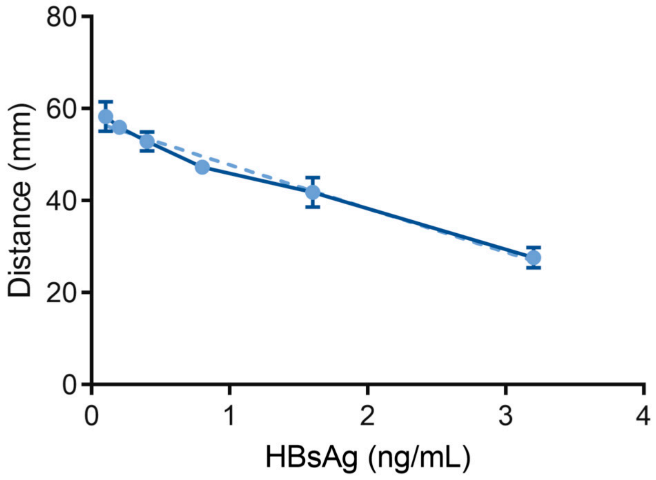

Figure S3. Linear response between the flow distance and the concentration of HBsAg using AHCS, mean+SD, n=3

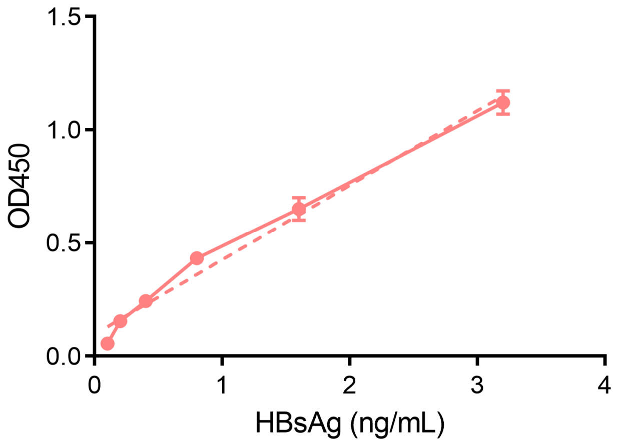

Figure S4. Linear response between the absorbance and the concentration of HBsAg using commercialized ELISA, mean+SD, n=3
